# Treatment Algorithms for Patients with Metastatic Non-Small Cell, Non-Squamous Lung Cancer

**DOI:** 10.3389/fonc.2014.00256

**Published:** 2014-09-29

**Authors:** Barbara Melosky

**Affiliations:** ^1^Medical Oncology, British Columbia Cancer Agency – Vancouver Centre, Vancouver, BC, Canada

**Keywords:** metatstatic non-squamous NSCLC, systemic therapy, chemotherapy, targeted therapy

## Abstract

A number of developments have altered the treatment paradigm for metastatic non-small cell, non-squamous lung cancer. These include increasing knowledge of molecular signal pathways, as well as the outcomes of several large-scale trials. As a result, treatments are becoming more efficacious and more personalized, and are changing the management and prognosis of non-small cell lung cancer patients. This is resulting in increased survival in select patient groups. In this paper, a simplified algorithm for treating patients with metastatic non-small cell, non-squamous lung cancer is presented.

## Treatment Paradigms

The previous standard of care in metastatic non-small cell lung cancer (NSCLC) was to treat patients with a platinum doublet for four to six cycles and to offer second-line therapy upon progression (1). The emergence of molecular testing, specifically for the epidermal growth factor receptor (EGFR) and for anaplastic lymphoma kinase (ALK), enables us to better tailor treatment strategies. The results from many recent large-scale clinical trials have validated these new treatment approaches.

Chemotherapy is still one of our most important weapons. Patients are now surviving longer. All patients should get three lines of therapy. With more treatment options becoming available, algorithms must be strategically designed to balance the need to give the best drugs first while ensuring that there are many more options available for later.

The treatment algorithm discussed in this chapter is based on Canadian recommendations. Although other health authorities may have different therapeutics available, basic principles still apply.

## First Treatment Decision Point: Histology and Mutation Testing

### Histology

In the past, the only histological criterion for therapeutic decision making was whether the lung cancer was small cell or non-small cell. The distinction between squamous or non-squamous cell histology became important and with the evolution of immunostaining, this distinction has become more evident. The reported incidence of squamous cell lung cancer has decreased over the last several decades (2), which may be due to natural phenomena or to the development of better immunostaining. For this same reason, the reported incidence of large cell, squamous, and non-small cell (otherwise unspecified) cancer is decreasing and the incidence adenocarcinoma is increasing. The emergence of more molecular tests is unlikely to lessen the importance of histology.

### Mutational testing

Mutation status influences the selection of first-line therapies. At this time, testing for EGFR mutations and for rearrangements in the ALK gene is recommended for patients with non-squamous histology. A number of initiatives are underway to help ensure that all advanced lung cancer patients will have mutation and biomarker testing available. Cooperation of all specialties is required, including respirologists, interventional radiologists, surgeons, and pathologists (3, 4).

Mutation profiles of cancer continue to rapidly evolve, especially for adenocarcinomas. As we better understand how other gene mutations influence lung cancer, mutation testing for other targets including MET, RET, and KRAS (5, 6) will become more likely and treatment algorithms will become even more complex.

## Treatment Options for Non-Squamous NSCLC

**Figure 1:**
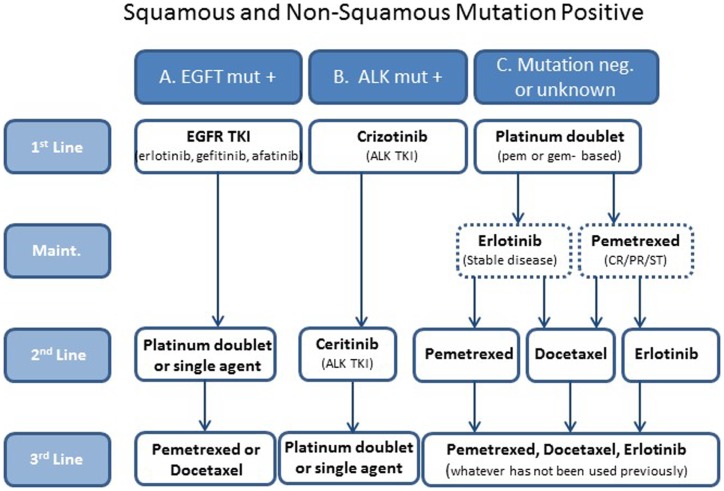
**A simplified treatment algorithm for patients with metastatic non-small cell non-squamous lung cancer**. Patients are divided into three groups based on histology and EGFR and ALK mutation status. Selection of therapies is based on these groups. The dashed boxes indicate that maintenance is optional in group C patients.

Histological analysis determines if patients have tumors with squamous or non-squamous histology. This chapter discusses non-squamous histology only. With mutation testing, patients can be divided into three groups: those whose tumors are positive for the EGFR mutation, which is 10–30% (6) (group A); those whose tumors are positive for the ALK mutation, approximately 5–7% (6) (group B); and those whose tumors do not have mutations in either EGFR or ALK or their mutation status is unknown, approximately 63–85% (group C). Therapy is selected based on these distinctions (Figure 1).

## Group A: Epidermal Growth Factor Receptor Mutation Positive

### First Line

Activity of EGFR is inhibited by tyrosine kinase inhibitors (TKIs), a unique class of orally administered, small molecule therapeutics that have found their way into the standard of care treatment in almost all types of malignancy. Several trials have demonstrated that TKIs, including erlotinib (7), gefitinib (8), and afatinib (9, 10), are efficacious first-line treatments for this patient population.

The efficacy of gefitinib was demonstrated in the IPASS trial, which compared first-line gefitinib with a carboplatin/paclitaxel doublet in an EGFR-unselected population. Although the gefitinib-treated patients demonstrated no increase in overall survival (OS), the time to progression (9.5 versus 6.3 months, respectively, HR 0.48; 95% CI 0.36 versus 0.64; *P* < 0.0001), overall response rate (71.2 versus 47.3%), and quality of life was improved in a subset of patients with EGFR-mutated tumors (8).

Erlotinib was shown to be advantageous in the first-line setting in the phase III EURTAC trial, where erlotinib-treated patients with EGFR mutation-positive tumors experienced progression free survival (PFS) of 9.7 months as compared to 5.2 months (HR = 0.37; *P* < 0.0001) in those patients treated with a platinum-based doublet such as docetaxel or gemcitabine (11). Response rate was 58% in the erlotinib arm versus 15% in the chemotherapy arm (*P* < 0.0001).

Afatinib has been shown to be superior to chemotherapy in the first line, in both the LUX-LUNG 3 (12) and the LUX-LUNG 6 (10) trials. LUX-LUNG3 was a phase III trial comparing afatinib versus chemotherapy (cisplatin/pemetrexed) as first-line treatments in chemo-naïve, NSCLC patients with EGFR mutation-positive tumors. LUX-LUNG 3 demonstrated that in the overall study population, median PFS was significantly longer with afatinib as compared to chemotherapy (11.1 versus 6.9 months; HR 0.58, 95% CI 0.43–0.78; *P* = 0.0004) (12). In patients with common EGFR mutation-positive tumors, median PFS was 13.6 versus 6.9 months on chemotherapy.

LUX-LUNG 6, a trial comparing afatinib with cisplatin/gemcitabine, confirmed that afatinib significantly improves PFS with a tolerable and manageable safety profile in Asian patients with advanced NSCLC who had tumors with EGFR mutations. In the overall study population, median PFS was significantly longer with afatinib as compared to chemotherapy (11.0 versus 5.6 months; HR 0.28, 95% CI 0.20–0.39; *P* < 0.0001) (10).

Selecting the TKI in this situation depends on many factors and is discussed in great detail elsewhere (13). Erlotinib and gefitinib are first generation TKIs, while afatinib and dacomitinib are second generation TKIs. Second generation TKIs differ from first generation. They block more ligands of the HER family. Perhaps more importantly, they are non-competitive inhibitors at the kinase site, so theoretically should prove to be more effective or confer a longer period to resistance than the first generation TKIs. We await the results and publication of several pivotal dacomitinib trials. Patient performance status, comorbidities, and age will all come into play in the decision making, as well as the availability of each therapeutic in a particular health authority. Unlike chemotherapy, TKIs can often be continued past progression in the lung cancer context, as long as there is a clinical benefit to the patient.

### Second-line therapy (first-line systemic therapy)

For all mutation-positive patients, the second-line therapy is the standard chemotherapy: a platinum doublet such as platinum/pemetrexed for four to six cycles (1). A single agent, such as pemetrexed, is an option for patients who are elderly or who may have a poor performance status and are not candidates for a platinum doublet. After second-line therapy, the patient is observed until progression.

### Third line therapy

Selection of third line therapy in these patients is straightforward; the single agent that has not been used so far. In most cases, this will be either docetaxel or pemetrexed to be continued until disease progression. After disease progression, patients with adequate performance status may be considered for clinical trials.

## Group B: Anaplastic Lymphoma Kinase Mutation Positive

### First-line therapy

Anaplastic lymphoma kinase gene rearrangements are found more commonly in adenocarcinomas than other types of lung cancers, and also found more commonly in light smokers or non-smokers. ALK gene rearrangements are thought to be exclusive of EGFR and KRAS mutations and occur in approximately 4–7% of lung cancers (6).

Patients with chromosomal rearrangements of the ALK gene have shown to have a stronger clinical response to crizotinib, an ALK-targeted TKI. A phase I trial in patients with advanced ALK-positive NSCLC demonstrated that crizotinib is associated with higher response rates and improved survival compared to that of crizotinib-naive controls (14), and as a result, received approval from FDA in the US and Health Canada in 2011 for use in this patient population.

Crizotinib was shown to be superior to standard chemotherapy in ALK mutation-positive pre-treated patients with advanced NSCLC (median PFS 7.7 months in the crizotinib group versus 3.0 months in the chemotherapy group (HR, 0.49; 95% CI, 0.37–0.64; *P* < 0.001); response rates 65% (95% CI, 58–72) for crizotinib versus 20% (95% CI, 14–26) with chemotherapy (*P* < 0.001) (15). Although this trial was conducted in pre-treated patients, using a drug that specifically inhibits the ALK pathway is perfect rationale to provide this treatment in the first-line. NCCN guidelines have recommended a first-line approach. Newly released results from the PROFILE 1014 phase III trial showed that crizotinib significantly prolonged PFS as compared to platinum-based chemotherapy in a first-line setting. (Available at: http://www.marketwatch.com/story/pfizer-reports-positive-phase-3-study-outcome-of-xalkori-crizotinib-compared-to-chemotherapy-in-previously-untreated-patients-with-alk-positive-advanced-non-small-cell-lung-cancer-nsclc-2014-03-25?reflink=MW_news_stmp. Accessed on April 4, 2014).

As with the other TKIs, crizotinib is often continued past progression as long as there is a clinical benefit to the patient.

### Second and third line therapy

Advanced NSCLC patients positive for the ALK mutation now have a new second-line agent (16). In April 2014, the FDA approved ceritinib for patients with ALK-positive NSCLC following treatment with crizotinib. The addition of this new ALK-targeted TKI into the ALK mutation-positive treatment paradigm pushes the use of a platinum doublet or single agent into the third line. As the treatment of ALK-positive patients evolves, we can expect treatment paradigms to continue to shift.

## Group C: Mutation Status Negative or Unknown

### First line

Patients with advanced NSCLC who have no known mutations in the EGFR or ALK genes or whose mutation status is unknown, receive the standard of care: a platinum doublet featuring pemetrexed or gemcitabine for four to six cycles. While there are many doublets to choose from in the first line including cisplatin/paclitaxel, cisplatin/gemcitabine, cisplatin/docetaxel, carboplatin/paclitaxel (1), the pivotal Scagliotti trial (17) demonstrated that patients with adenocarcinoma fare better with cisplatin/pemetrexed than cisplatin/gemcitabine in the first line (OS 12.6 versus 10.9 months; HR, 0.84; 95% CI, 0.71–0.99; superiority *P* = 0.033).

### Another decision point: Maintenance after first line

Maintenance therapy is the focus of another article in this journal (18). This therapeutic approach is important enough that it will be addressed in this article as well, albeit briefly.

Maintenance therapy in NSCLC is defined as a therapeutic agent that is administered after completion of the first line, but before the disease progresses. Results suggest that NSCLC patients may be more likely to receive additional therapy if maintenance is offered immediately after front-line therapy, before progression occurs (19–21). A recent meta-analysis of 13 maintenance chemotherapy trials demonstrated an improvement in PFS and in OS in patients who had experienced maintenance therapy (22). The most promising strategies involved administering an approved second-line NSCLC therapeutic for maintenance therapy (23, 24).

There are two types of maintenance therapy to consider, continuous and switch maintenance. Continuous maintenance is when patients are offered one of the agents in the induction doublet to be continued after first-line therapy until progression. This is an option for patients who have not progressed on first line. The PARAMOUNT trial demonstrated that pemetrexed maintenance given to NSCLC patients with tumors having non-squamous histology after first-line platinum/pemetrexed had a significantly reduced risk of disease progression over placebo (20). Switch maintenance, also referred to as “early second line,” is when a new agent is given after the completion of four cycles of first-line-doublet. Studies have shown that both pemetrexed (19) and erlotinib (21) improve both PFS and OS when administered as maintenance therapy after first-line chemotherapy is completed.

To ask our patients to take maintenance therapy requires careful discussion and consideration. Residual nausea, fatigue, and alopecia from chemotherapy can take time to resolve, and many patients may choose to have drug holiday after 3–4 months of a platinum regimen. Many may refuse maintenance therapy as it requires monitoring visits in addition to treatment. Patients who decline maintenance therapy should be observed closely until progression so that they may receive another line of therapy.

### Second line

Proven second-line options for patients whose tumors are mutation negative or mutation unknown, include docetaxel (23), erlotinib (25), and pemetrexed (26). Pemetrexed can only be offered if it was not used in first-line or maintenance therapy. If a pemetrexed platinum doublet was selected in the first line or for maintenance, docetaxel or erlotinib is selected for the second line.

The BR-21 trial demonstrated that erlotinib prolongs survival in patients with NCSLC following the failure of first-line or second-line chemotherapy (25). This multicenter, randomized, controlled, Phase III study randomized patients who had failed first- or second-line chemotherapy to either erlotinib or to placebo. Patient selection was not based on EGFR status, gender, smoking history, or type of NSCLC. The study met its primary endpoint of improving OS (median OS of 6.7 versus 4.7 months (HR, 0.70; 95 CI, 0.58–0.85; *P* < 0.001), and demonstrated statistically significant effects in secondary endpoints including PFS, time to symptom deterioration, and response rate. Overall, 8.9% of patients achieved an objective response to erlotinib (*P* < 0.001); the median duration of response was 34.2 weeks. This trial demonstrated a survival benefit in all patients regardless of EGFR mutation status or histology (25). Although still controversial, BR-21 led to an EGFR TKI to become standard of care in second and third line in unselected patients with NSCLC.

### Third line

Third line therapies for mutation negative or mutation unknown patients may include whatever agents were not given in previous lines. This may include docetaxel (23), erlotinib (25), and pemetrexed (26). A significant limitation of therapy selection is that few trials have tested these different agents in later therapy, and sequences and combinations of these therapies have not been tested. Third line therapy is continued until disease progression or undue toxicity. After disease progression patients with adequate performance status may be considered for clinical trials.

## Conclusion

**Figure 2:**
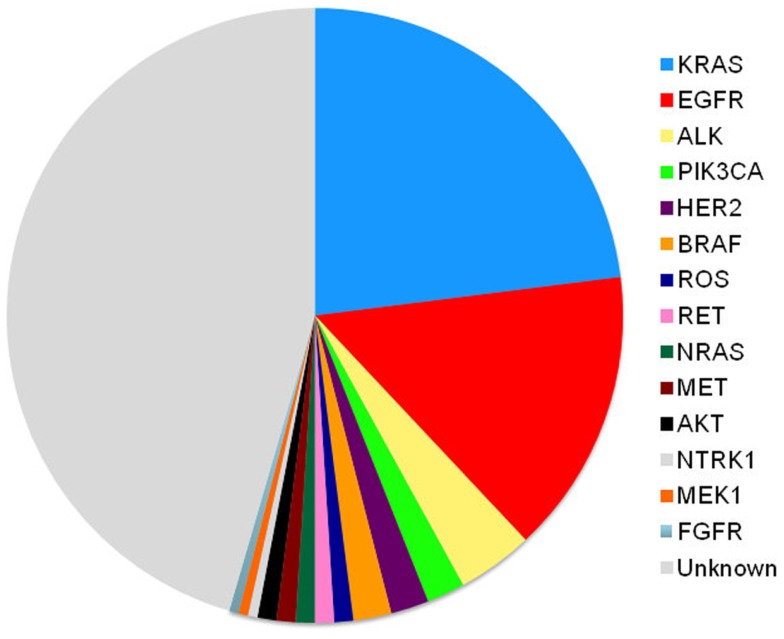
**Oncogenic drivers in lung adenocarcinoma (27–29)**.

Although we test for EGFR and ALK mutations and have treatments for those patients, therapy is still palliative in nature. Chemotherapy still remains our therapeutic backbone. However, the treatment algorithm will always be changing. As we continue to define the drivers of thoracic malignancy (Figure 2), our discovery and understanding of mutations in non-squamous, NSCLC will evolve. We will combine different targeted agents to overcome the development of resistance and will learn about the best ways to sequence these agents. Physicians should aim to provide three lines of therapy to patients. The discovery of new molecular targets and the development of targeted therapy ultimately benefit the patients with NSCLC.

## Conflict of Interest Statement

The author declares that the research was conducted in the absence of any commercial or financial relationships that could be construed as a potential conflict of interest. The Review Editor Vera Hirsh declares that, despite having collaborated with author Barbara Melosky, the review process was handled objectively and no conflict of interest exists.

## Abbreviations

ATP, adenosine triphosphate; ALK, anaplastic lymphoma kinase; EGFR, epidermal growth factor receptor; HER 2, human epidermal growth factor receptor 2; NCI, National Cancer Institute; NSCLC, non-small cell lung cancer; RCT, randomized clinical trial; OS, overall survival; PFS, progression free survival; TKI, tyrosine kinase inhibitor.
